# Evaluating Rates and Determinants of COVID-19 Vaccine Hesitancy for Adults and Children in the Singapore Population: Strengthening Our Community’s Resilience against Threats from Emerging Infections (SOCRATEs) Cohort

**DOI:** 10.3390/vaccines9121415

**Published:** 2021-11-30

**Authors:** Konstadina Griva, Kevin Y. K. Tan, Frederick H. F. Chan, Ramanathan Periakaruppan, Brenda W. L. Ong, Alexius S. E. Soh, Mark IC. Chen

**Affiliations:** 1 Population/Global Health, Lee Kong Chian School of Medicine, Nanyang Technological University, Singapore 308232, Singapore; kevin.tanyk@ntu.edu.sg (K.Y.K.T.); huifei001@e.ntu.edu.sg (F.H.F.C.); rama0072@e.ntu.edu.sg (R.P.); 2 Infectious Disease Research and Training Office, National Centre for Infectious Diseases, 16 Jln Tan Tock Seng, Singapore 308442, Singapore; brenda_ONG@ncid.sg (B.W.L.O.); alexius_matthias_se_soh@ncid.sg (A.S.E.S.); mark_ic_chen@ncid.sg (M.I.C.)

**Keywords:** vaccine hesitancy, parental hesitancy, COVID-19, sociodemographic factors, psychosocial factors

## Abstract

COVID-19 vaccines are crucial for achieving sufficient immunisation coverage to manage the pandemic, but vaccine hesitancy persists. This study aimed to investigate the prevalence and determinants of vaccine hesitancy in adults and in parents for vaccinating their children using an integrated social cognition model. A community-based cohort in Singapore [*N* = 1623] completed a survey (wave 25) between June and July 2021 which measured their risk perceptions, distress, trust, vaccination beliefs, and vaccine intentions/behaviours. Results indicated low rates of hesitancy (9.9%) for own vaccination, with most concerns citing side effects, safety, and hasty development. Remaining respondents were vaccinated (69%) or intended to vaccinate (21%). The multivariable model (non-vaccinated respondents) indicated that, living with people in poor health, subjective norm, moral norm, benefits, and necessity of vaccination were associated with lower vaccine hesitancy (*R*^2^ Cox & Snell: 51.4%; *p* < 0.001). Hesitancy rates were higher for children’s vaccination (15.9%), with male gender, lower perceived vaccine benefits, high COVID-19 risk perceptions, vaccination concerns, and necessity beliefs associated with higher odds of parental vaccine hesitancy (*R*^2^ Cox & Snell = 36.4%; *p* < 0.001). While levels of vaccine acceptance are high, more targeted messages are needed. For adults’ vaccination, more emphasis should be on benefits and social gains, while for parental hesitancy, messages related to safety should be prioritised.

## 1. Introduction

As of August 2021, the COVID-19 pandemic saw over 200 million positive cases worldwide and over 60,000 people in Singapore infected [1,2]. With COVID-19 vaccines reportedly able to effectively reduce the spread and severity of the disease [3,4,5], vaccination has been widely considered a key preventive measure in infection control, disease eradication, and in reducing mortality and morbidity rates [6,7,8]. The vaccination program in Singapore was launched in December 2020 and initially prioritised individuals considered at high risk for COVID-19 (i.e., frontline healthcare /community workers, elderly aged >60 years) [9,10]. The program was expanded to all adults [11,12] and on 11 June 2021, children/youth aged 12 years of age and above were invited to take the COVID-19 vaccines [12,13]. As of June 2021, the Ministry of Health (MOH) of Singapore had authorised two COVID-19 mRNA vaccines for administration under its National Vaccine Programme: Comirnaty (by Pfizer–BioNTech; Pfizer, New York, US; BioNTech, Mainz, Germany) [14] and Moderna (by Moderna Inc., Cambridge, MA, USA) [15]. The ministry detailed the severity of the side effects of the mRNA vaccines as mild, with common symptoms such as fatigue, headaches, chills and muscle and joint pains expected to subside within a few days [16]. Adverse events related to the mRNA vaccines were reportedly low, and accounted for only 0.12% of all administered doses in Singapore as of July 2021 [17]. Despite the timely provision and accessibility of COVID-19 vaccines in Singapore and low adverse event incidence rate, vaccine hesitancy could undermine uptake rates for main and booster vaccination and result in insufficient immunisation coverage against COVID-19.

Vaccine hesitancy is defined as the delay in acceptance or refusal of vaccination despite availability of vaccination services [18]. Mistrust related to the novelty of mRNA technology [19] and its rapid approval and rollout of vaccination programs [20] had been reported globally, and has fuelled anti-vaccine movements [21]. While vaccine uptake had been increasing steadily in high resource/income settings, COVID-19 vaccine hesitancy rates are still substantial, ranging from 10–50% across various settings [22,23,24,25,26,27,28,29,30,31,32,33,34,35,36,37,38,39,40,41,42] (e.g., 28.8% in France [23], 65.0% in Portugal [27], 49.2% in Poland [42], 30.0% in New Zealand [41], 43.9% in Japan [22] and 22.0% in the US [25]). A report in Singapore conducted in March 2021 showed that vaccine hesitancy rate was 33.0% [43].

While Singapore ranks highly on vaccination targets, hesitancy persists for a considerable proportion of the population and its determinants are not well understood. With the booster vaccination program underway, it is essential to identify drivers of hesitancy, so as to address public concerns, increase confidence and bolster vaccine uptake. There are multiple factors, both individual and system-level, relevant to vaccine hesitancy [18,44]. As concluded by the World Health Organisation Strategic Advisory Group of Experts, these include (a) sociodemographic and institutional features, (b) individual and social group influences and (c) vaccine specific issues like mode of administration [18,44]. Of particular interest are the potentially modifiable psychological determinants of vaccine hesitancy. Studies that applied Social Cognition Models [45,46,47] in context of COVID-19 vaccination indicate that such intentions and behaviours are reasoned processes that are determined by beliefs such as risk perceptions [22,23,26,28,30,31,32,33,35,39,48], vaccination attitudes (i.e., benefits [22,26,28,29,31,32,35,36,37,39,40,41,48], concerns [22,23,27,28,30,32,33,34,35,36,37,39,41,48], necessity [29,30,33]), social norms [30,36,37], moral norms [22,29,32,48] and perceptions of institutional trust [20,22,24,27,30,33,36,37]. While the rapidly emerging research on COVID-19 vaccine hesitancy provides empirical support for such individual and social processes, these parameters are often studied in isolation or as limited sets of psychosocial parameters [20,26,31,33,34,35,39,40,41,48]. Research on parental hesitancy of COVID-19 vaccination for children is also scarce [32,48,49] and the drivers of parental hesitancy are not well understood as the focus has primarily been on sociodemographic parameters. Parental hesitancy rates were noted to range between 11% and 35% [32,48,49], and noted to be higher for parents from ethnic minority groups (relative to Whites), lower household income [32,49], with more children [32] and when the child has a chronic illness [48]. With vaccination programs now focusing on children, it is important to elucidate the different drivers of vaccine hesitancy in relation to own (adult) vaccination as well as vaccination for children, with emphasis on psychosocial parameters that are amenable to change.

This study sets out to address these gaps. Using an integrated psychosocial model based on theoretical review and prior vaccination studies and data from a nationally representative population cohort, this study sought to evaluate and contrast rates and determinants of COVID-19 vaccine hesitancy for adult and children vaccination. The aims of this study are threefold: (1) To document the rates of COVID-19 vaccine hesitancy in adults in Singapore focusing on those that have yet to take the vaccine; (2) to determine the hesitancy rate for parents in vaccinating their children between 12 and 18 years old as part of the newly launched children vaccination program; and (3) to identify sociodemographic and psychosocial factors associated with vaccine hesitancy towards own and children COVID-19 vaccination.

## 2. Materials and Methods

### 2.1. Study Population and Setting

Participants from the SOCRATEs (Strengthening Our Community’s Resilience Against Threats from Emerging infections) epidemiological cohort in Singapore were surveyed between June and July 2021 (wave 25 of data collection). SOCRATEs is a community-based study cohort established to assess the awareness, knowledge and perceptions of the public on infectious disease outbreaks in Singapore [43] with recurrent waves of data collection conducted rapidly. The current survey (wave 25) opened shortly after the launch of the COVID-19 vaccination program for children aged 12–18 years old. At that time, Singapore was in Phase 2 (Heightened Alert) [50]. In terms of COVID-19 epidemiological data, at closure of the survey in end July 2021, Singapore had recorded 64,981 COVID-19 infections and 37 deaths [51], and achieved a vaccination completion rate of 59% [52]. During the study window, COVID-19 vaccination was not mandated but highly recommended and offered free for the community. No vaccination-differentiated measures were in force.

Sampling was conducted using door-to-door recruitment, social media posts, self-referral and by recruiting past participants from the HELIOS study cohort. HELIOS (Nanyang Technological University ethics approval IRB–2016–11–030) is a prospective longitudinal population cohort comprising the multi-ethnic Asian population of Singapore in which participants were recruited through a range of community outreach programmes to ensure participation from ethnic minorities, working age and lower socioeconomic groups to reflect Singapore’s national statistics. Door-to-door recruitment comprised of an equal number of household units that were randomly selected across five geographical zones in Singapore, with a limit of four participants per household. Snowball sampling was used to recruit participants through social media. Telephone-based surveys were conducted every 2 to 3 months for participants who were unable to access or complete the online survey form. Participants had to be (1) Singaporeans or Permanent Residents (PR), (2) 16 years old or above, (3) residing in Singapore and (4) were able to use a digital device to access the online survey. A total of 1623 participants (door-to-door = 115 [7.1%], social media and self-referral = 849 [52.3%], HELIOS = 659 [40.6%]) responded to the survey, which amounted to a response rate of 84.5%. The resulting sample constituted approximately 0.03% of the total Singapore population in June 2021 [53], and represented well with the national registry [54]. The study was approved by the National Healthcare Group (NHG) Institutional Review Board.

### 2.2. Measures

Sociodemographic information including age, gender, race, education, employment, housing, household income, and whether participants were living with children, spouse, or with people in poor health was collected. Clinical information about the health of the participants, such as their experience with COVID-19, daily regular contact, and chronic conditions were also obtained. 

To screen for depression, the patient health questionnaire-2 (PHQ-2) tool was used [55,56]. The generalised anxiety disorder-2 (GAD-2) tool was used to screen for anxiety [57,58].

### 2.3. Psychosocial Factors 

A set of multi-item self-report measures were developed with reference to prior research on vaccination to assess the following psychosocial constructs from relevant models such as the health belief model [35,36,37,38,39,40,59,60], the theory of planned behaviour [45,46,61] and social cognitive theory [45,46,47]: perceived risk of COVID-19 (including items involving perceived susceptibility and severity of COVID-19 infection) (5-items); perceived benefits (6-items); COVID-19 vaccination concerns (5-items); trust (3-items); subjective norm (3-items); moral norm (3-items); necessity of the vaccine (2-items). Items were rated on a 5-point Likert scale ranging from 1 indicating ‘Strongly agree’ to 5 ‘Strongly disagree’. After reverse-coding, higher scores signified higher perception of risk, benefits, concerns, trust, norms, and necessity.

The items were reviewed by experts with clinical and behavioural health expertise (KG, MC, AS) to assure quality, tested with two members of public to ensure clarity and comprehension but extensive piloting was not done due to the critical nature of the survey.

### 2.4. Intention to Vaccinate against COVID-19 

For own intentions, participants were asked on their intention to get vaccinated. Response options were ‘Already vaccinated’, ‘Yes’, ‘No’ or ‘Undecided’, with the latter two considered as ‘vaccine-hesitant’ responses. 

Participants with children aged 12–18 were also asked on their intention to vaccinate their children. Response options included ‘Yes’, ‘No’, ‘Unsure’ and ‘Prefer to wait’, with the latter three indicating hesitancy to vaccinate their children.

### 2.5. Data Analysis

Two multivariable binary logistic regressions (via enter method) were conducted to identify the sociodemographic and psychosocial predictors of the study outcomes: own vaccine hesitancy and parental hesitancy on children’s vaccination. The model to predict own hesitancy only included participants who had not yet been vaccinated. The regression to predict parental hesitancy of children’s vaccination was run only for participants with children aged 12–18. For ordinal and nominal variables in both models, the group with the highest frequency of respondents was chosen as the reference. Reliability analysis was conducted, and the internal consistency of all measures were deemed acceptable (Cronbach’s alpha > 0.6) [62].

## 3. Results

The final survey sample consisted of 1623 participants (60.8% female, mean age = 45.7 years, response rate = 84.5%). Participants were predominantly Chinese (89.5%), employed (57.8%), had a post-secondary education or higher (86.0%), resided in 4–5 room HDB flats, DBSS/HUDC housing or executive apartments (59.6%) and had a monthly household income between S$5,000 and S$12,999 (50.2%). The subset of responders with children between 12 and 18 years old had a similar profile. They were mostly female (mothers) (59.2%), Chinese (87.1%), aged 41 to 50 years old (49.8%) (mean age = 46.3 years, *SD* = 2.58), with post-secondary education or higher (83.7%), employed (59.7%) and had a monthly household income of S$5,000 to S$12,999 (57.1%) (Table 1).

### 3.1. Vaccine Hesitancy Rate

Of the 1623 respondents, 69% (*n* = 1120) had already received the COVID-19 vaccine, 21% (*n* = 341) intended/wanted to be vaccinated, 2.6% (*n* = 43) refused vaccination and 7.3% (*n* = 119) remained undecided. The overall vaccine hesitancy rate (refused and undecided) was 9.9% (*n* = 162). Those who had taken or intended to take the vaccine indicated the following reasons for their decision: return to normalcy (73.9%, *n* = 1079); worry over community infection (61.9%, *n* = 904) and to resume travel (50.2%, *n* = 733). Respondents who did not want to be vaccinated or were undecided cited the following top three reasons for their decision: concerns about side effects (refused = 88.4%, *n* = 38; undecided = 81.5%, *n* = 97), rushed vaccine development (refused = 65.1%, *n* = 28; undecided = 66.4%, *n* = 79) and preference to wait and ensure vaccine safety (refused = 34.9%, *n* = 15; undecided = 64.7%, *n* = 77).

To identify correlates of own vaccine hesitancy among the subset of those not vaccinated (*n* = 503), comparisons were undertaken between those who intended to take the vaccines (‘no vaccine hesitancy group’) and those who refused or were undecided (‘vaccine hesitant group’). Significant differences were noted in gender, age, household income, occupation and having a chronic condition (Table 1). These indicate that vaccine hesitancy was higher for female, employed respondents, aged 31 to 40 years old (mean age = 35.4 years, SD = 2.77), were earning an income between S$5,000 and S$12,999 and had no chronic illnesses. With regards to psychosocial parameters, significant differences were shown for perceived risk of COVID-19, trust, subjective norm, benefits, moral norm, concern, and necessity variables between those with vaccine hesitance and those not hesitant (Table 2). Participants with vaccine hesitancy reported lower risk perception of COVID-19, were more distrustful and concerned about the vaccine, perceived less benefits and necessity for the vaccine and reported lower moral and subjective norms about the vaccine compared to participants who were willing to be vaccinated.

### 3.2. Factors Affecting Vaccine Hesitancy

Binary logistic regression on the subset of participants who have yet to be vaccinated (*n* = 503) indicated that individuals who were living with people in poor health were more likely to be less hesitant about receiving the vaccine themselves. Moreover, individuals who perceive the vaccine as beneficial and necessary for themselves possessed lower odds of vaccine hesitancy. Adherence to subjective and moral norms also predicted a decreased likelihood of vaccine hesitancy. The binary logistic regression model was statistically significant, χ^2^(32) = 361.974, *p* < 0.001, and accounted for 51.4% (Cox & Snell) to 71.8% (Nagelkerke) of the variance in adult vaccine hesitancy (Table 3).

### 3.3. Parental Vaccine Hesitancy Rate

Of the 238 participants with children aged 12–18, a total of 233 reported their intention to vaccinate or not vaccinate their children. The majority indicated that they would proceed with vaccination (84.1%, *n* = 196—no vaccine hesitancy group), while the remaining either refused (3.9%, *n* = 9), were unsure (4.7%, *n* = 11) or preferred to wait (7.3%, *n* = 17), thus signifying an overall vaccinate hesitancy rate of 15.9%. Among the psychosocial variables, significant differences in trust, moral norm, benefits, subjective norm, concern, anxiety, and necessity were observed (Table 2). Compared to parents who were willing to vaccinate their children, those who were hesitant to do so had lower mean scores for trust, subjective norm, moral norm, anxiety, benefits and necessity for the vaccine but reported significantly higher concerns about the vaccine. None of the sociodemographic parameters differed between subgroups (Table 1).

### 3.4. Factors Affecting Parental Vaccine Hesitancy

The multivariable binary logistic regression model accounted for 36.4% (Cox & Snell) to 62.4% (Nagelkerke) of the variance in parental vaccine hesitancy, χ^2^(33) = 105.497, *p* < 0.001. The following parameters were shown to be significant: gender (male), benefits of the vaccine, perceived personal necessity for the vaccine, concerns about the vaccine and the perceived risk of COVID-19 (Table 3). The odds for parental vaccine hesitancy for children vaccination were higher for male (father), individuals with lower risk perception of COVID-19, lower perceived benefits of the vaccines, higher vaccination concerns and perceptions of higher personal necessity for the COVID-19 vaccine.

## 4. Discussion

Using data from the SOCRATEs national epidemiological cohort in Singapore, we observed that the prevalence of vaccine hesitancy was low. With regards to adults, vaccine hesitancy was only 9.9% at 6 months post-launch of the adult vaccination program, which compares favourably with rates reported in other settings, (e.g., Japan (43.9%) [22], Poland (49.2%) [42] and New Zealand (30%) [41]) which had similar epidemiological COVID-19 profiles and pandemic responses to Singapore.

Parental hesitancy for children (12–18 years) vaccination was similarly low (15.9%) even though the vaccination program was launched only one week prior to data collection. Prior work indicated higher rates of parental hesitancy (e.g., South Korea (36%) [63], Australia (24%) [64] and USA (25%) [48]), but this may be due to the fact that these studies also recruited parents of younger children (0–12 years), for whom vaccination authorisation was still pending.

Using an integrated psychosocial model informed by relevant theoretical and empirical work, the study findings indicated that both individual and social factors are critical determinants of vaccine hesitancy (own decision) as well as hesitancy when making decision for children’s vaccination, but specific predictors differed. Adult vaccine hesitancy was driven by vaccination beliefs [i.e., necessity of the vaccine, vaccine benefits for self and wider community (moral norm)], social/peer influence (subjective norms) and living with people in poor health, which were in line with prior work [29,37,39,65].

As individuals in poor health are at higher risk for poor prognosis if infected with COVID-19 [65], their vulnerability could have invoked an increased sense of responsibility and accountability in people living with them, resulting in increased willingness to receive the vaccine. Relatedly, collective responsibility as reflected in moral norms was also shown to be associated with lower odds of hesitancy, consistent with prior work [29,66]. Messages leveraging and emphasising social responsibility may be particularly effective strategies to increase vaccine acceptance especially for collectivism-orientated settings such as Singapore where the value of familism is highly endorsed. Aligned with collectivism, social influence from family members or friends (i.e., social norms) also predicted adult vaccine hesitancy, consistent with previous research [67,68,69]. Interestingly, COVID-19 risk perception and vaccination concerns, although significant at binary analyses, when combined with beliefs about ‘value’ of vaccination (benefits, personal necessity, collective/moral value), were no longer significant. Singapore’s response has been largely effective in terms of limiting case numbers and fatality rates, hence perceived risk may be lower and hence less important than perceptions of benefits and social value. It is also important to note that data in our study were also collected several months into the implementation of adult COVID-19 vaccination programs whereas prior studies that identified risk perceptions and vaccination concerns as key determinants of vaccine hesitancy were conducted in 2021 before the rollout of COVID-19 vaccinations [29,37,39]. Risk perception and vaccination concerns are likely to be higher at early stages of the vaccination program compared to later stages when cases stabilise and vaccinations become available, and thus their importance in predicting vaccine hesitancy may reduce over time. Health communications about vaccinations may therefore need to gradually shift focus from addressing risk perceptions and concerns towards emphasising vaccination benefits for the individual and the community.

In contrast to adult vaccine hesitancy, parental hesitancy for children vaccination was associated with both COVID-19 beliefs (risk perceptions) and vaccination beliefs both in terms of vaccination benefits and concerns. As the vaccination program for children was only launched one week prior to data collection [13], vaccine concerns related to safety are likely to remain especially prominent [22,23,27,28,30,32,33,34,35,36,37,39,41,48,70]. It is possible that over time with the progression of children COVID-19 vaccinations, concerns may wane, and perceptions of value and benefits of vaccination for children may acquire more weight, as shown with adults’ own vaccination. To increase parents’ vaccine confidence and vaccine uptake for children, it is hence important to address both perceptions of harm/threat in tandem with perceptions of benefits.

Parental hesitancy was higher for those not yet vaccinated and for males (fathers) than females (mothers), which was consistent with other studies that found gender differences in parental vaccine hesitancy [71,72]. The gender effect may be explained by the fact that women are more likely to have more health encounters and consultations, and hence greater exposure to health communication about vaccines [73], and are more likely to be health advocates or decision-makers for other family members including children [74].

Patterns of results indicated that health policies and programs could leverage on the high level of vaccine acceptance, and responsively adapt vaccination messages to audiences and towards stages of program rollout or implementation. It is important to address vaccine concerns around safety and efficacy especially when new COVID-19 vaccine initiatives and programs are launched (e.g., younger children, booster programs). Reports of risk of adverse events related to COVID-19 vaccination, even if low, may fuel mistrust or fear over vaccine safety [75,76,77]. The occurrence of innocuous and transient side effects, such as fatigue, body aches and low-grade fever, which are normal signs of vaccine reactogenicity may also undermine vaccine acceptance especially for healthy children and youth that are not among the high-risk groups for severe COVID-19. Communication about vaccine safety should hence target both adverse events and side effects signalling vaccine reactogenicity to boost vaccine confidence and vaccine update. However, addressing only concerns may be insufficient in eliminating hesitancy. Focus may also need to shift increasingly towards benefits when these programs are established. Measures that promote a sense of civic responsibility could be useful in capitalising on the social influences surrounding vaccine hesitancy.

The current study has several limitations and strengths to consider. First, the study reported on cross-sectional data, hence no causal inferences can be made. The study sample also recruited from an ethnically diverse setting comprising predominantly individuals of Asian ancestries. While the study’s profile was representative of the national registry in Singapore [54], results may not be readily generalised to other more homogenous, non-Asian settings without replication. It is also important to note that although the overall sample size was large, the numbers of those reporting vaccine hesitancy were modest, hence separate analyses could not be conducted for those undecided/ambivalent and those who refused the COVID-19 vaccination. More work is warranted to better understand what drives ambivalence and what may drive antivaccination attitudes and decisions. Finally, as the pandemic evolves over time and pandemic or vaccination-related policies change, longitudinal studies are required to understand the changes in vaccine attitudes, intentions and behaviours and vaccination hesitancy trajectories or profiles over time.

## 5. Conclusions

While COVID-19 vaccine hesitancy rates in Singapore are low, there were sociodemographic and psychosocial factors that contribute towards vaccine hesitancy in Singapore, and were found to largely differ depending on the vaccination target. These factors could serve to inform strategies in promoting effective and adaptive health communication tactics towards vaccination intention that concern oneself and others, and to devise programs that could further resolve doubts about the vaccine. Addressing hesitancy in these areas could nudge undecided individuals to take the vaccine and provide Singapore with a higher immunisation coverage against COVID-19.

## Figures and Tables

**Table 1:** Sociodemographic characteristics of participants (*n* = 1623) and their associated vaccine hesitancy rates.

**Sociodemographic** **Characteristics** **[*n* (%)]**	**Total** ***n*** ** = 1623 (%)**	**Unvaccinated** ***n*** ** = 503 (%)**				**Parents with Children Aged 12 to 18 Years** ***n*** ** = 233 (%)**			
		**Willing to Get** **Vaccinated**	**Hesitant**	***p*** **-Value**	**Cramer’s** **V**	**Willing to Vaccinate Child**	**Hesitant**	***p*** **-Value**	**Cramer’s V**
**Total**	1623 (100.0)	341 (67.8)	162 (32.2)			196 (84.1)	37 (15.9)		
**Gender**				0.001	0.15			0.485	0.05
Male	636 (39.2)	144 (42.2)	43 (26.5)			78 (39.8)	17 (45.9)		
Female	987 (60.8)	197 (57.8)	119 (73.5)			118 (60.2)	20 (54.1)		
**Race**				0.052	0.09			0.683	0.03
Chinese	1452 (89.5)	301 (88.3)	152 (93.8)			170 (86.7)	33 (89.2)		
Non-Chinese	171 (10.5)	40 (11.7)	10 (6.2)			26 (13.3)	4 (10.8)		
**Age**				0.024	0.15			0.680	0.10
17–30	322 (19.8)	82 (24.0)	25 (15.4)			30 (15.3)	4 (10.8)		
31–40	319 (19.7)	103 (30.2)	39 (24.1)			14 (7.1)	5 (13.5)		
41–50	329 (20.3)	57 (16.7)	31 (19.1)			97 (49.5)	19 (51.4)		
51–60	314 (19.3)	45 (13.2)	34 (21.0)			46 (23.5)	8 (21.6)		
61+	339 (20.9)	54 (15.8)	33 (20.4)			9 (4.6)	1 (2.7)		
**Mean Age (*SD*)**	45.7 (15.0) ^1^	42.0 (14.6) ^1^	46.4 (14.3) ^1^	0.001	0.31^2^	44.5 (11.5) ^1^	45.4 (10.3) ^1^	0.671	0.08 ^2^
**Highest** **Education**				0.881	0.01			0.616	0.03
Secondary Education or lower	227 (14.0)	48 (14.1)	22 (13.6)			33 (16.8)	5 (13.5)		
Post-secondary education or higher	1396 (86.0)	293 (85.9)	140 (86.4)			163 (83.2)	32 (86.5)		
**Monthly Household** **Income ^3^**				0.003	0.15			0.771	0.05
Less than S$5,000	506 (31.2)	96 (28.2)	65 (40.1)			33 (16.8)	8 (21.6)		
S$5,000–S$12,999	813 (50.2)	170 (50.0)	79 (48.8)			115 (58.7)	18 (54.1)		
More than SGD$13,000	302 (18.6)	74 (21.8)	18 (11.1)			48 (24.5)	9 (24.3)		
**Housing Type**				0.686	0.04			0.952	0.02
1–3 room HDB	211 (13.0)	44 (12.9)	25 (15.4)			18 (9.2)	4 (10.8)		
4–5 room HDB/ Executive Apartment/ DBSS/HUDC	967 (59.6)	209 (61.3)	99 (61.1)			114 (58.2)	21 (56.8)		
Condominium/ Landed Property	445 (27.4)	88 (25.8)	38 (23.5)			64 (32.7)	12 (32.4)		
**Occupation**				0.002	0.17			0.849	0.06
Employed	938 (57.8)	211 (61.9)	83 (51.2)			119 (60.7)	20 (54.1)		
Schooling	154 (9.5)	40 (11.7)	10 (6.2)			22 (11.2)	4 (10.8)		
Self-employed	190 (11.7)	38 (11.1)	30 (18.5)			24 (12.2)	6 (16.2)		
Not employed or schooling	341 (21.0)	52 (8.8)	39 (4.9)			31 (15.8)	7 (18.9)		
**Daily Regular Contact**				0.414	0.08			0.766	0.07
Less than 10 people	802 (49.4)	165 (48.4)	87 (53.7)			86 (43.9)	18 (48.6)		
10–19 people	384 (23.7)	88 (25.8)	41 (25.3)			45 (23.0)	10 (27.0)		
20–49 people	280 (17.3)	58 (17.0)	26 (16.0)			37 (18.9)	5 (13.5)		
50 people or more	157 (9.7)	30 (78.9)	8 (21.1)			28 (14.3)	4 (10.8)		
**Living with children aged** **0 to 12 years**				0.252	0.05			0.722	0.02
No	1333 (82.1)	258 (75.7)	130 (80.2)			133 (67.9)	24 (64.9)		
Yes	290 (17.9)	83 (24.3)	32 (19.8)			63 (32.1)	13 (35.1)		
**Living with youth aged** **12 to 18 years**				0.439	0.03			0.338	0.06
No	1385 (85.3)	297 (87.1)	145 (89.5)			21 (10.7)	6 (16.2)		
Yes	238 (14.7)	44 (12.9)	17 (10.5)			175 (89.3)	31 (83.8)		
**Living with people with poor health**				0.354	0.04			0.287	0.07
No	1406 (86.6)	286 (83.9)	141 (87.0)			176 (89.8)	31 (83.8)		
Yes	217 (13.4)	55 (16.1)	21 (13.0)			20 (10.2)	6 (16.2)		
**Living with people** **vulnerable to COVID-19**				0.229	0.05			0.345	0.06
No	1249 (77.0)	264 (77.4)	133 (82.1)			169 (86.2)	34 (91.9)		
Yes	374 (23.0)	77 (22.6)	29 (17.9)			27 (13.8)	3 (8.1)		
**Living with spouse**				0.279	0.05			0.931	0.01
No	880 (54.2)	191 (56.0)	99 (61.1)			65 (33.2)	12 (32.4)		
Yes	743 (45.8)	150 (44.0)	63 (38.9)			131 (66.8)	25 (67.6)		
**Has a Chronic Condition**				0.022	0.10			0.879	0.01
No	1185 (73.0)	264 (77.4)	110 (67.9)			146 (74.5)	28 (75.7)		
Yes	438 (27.0)	77 (22.6)	52 (32.1)			50 (25.5)	9 (24.3)		

^1^ Values outside of brackets represent the mean age while values inside brackets represent standard deviation. ^2^ Effect size is in Cohen’s *d*. ^3^ Monthly household income contains missing data from two participants, who were excluded from binary logistic regression

**Table 2:** Mean scores and standard deviation of all participants on psychosocial, anxiety and depression variables.

Psychosocial Characteristics [*M* (*SD*)]	Total *n* = 1623 (*SD*)	Unvaccinated *n* = 503 (*SD*)				Parents with Children Aged 12 to 18 Years *n* = 233 (*SD*)			
		Willing to Get Vaccinated	Hesitant	*p*-Value	Cohen’s *d*	Willing to Vaccinate Child	Hesitant	*p*-Value	Cohen’s *d*
**Perceived risk of COVID-19**	3.37 (0.48)	3.59 (0.61)	3.40 (0.86)	0.014	0.27	3.54 (0.67)	3.37 (0.61)	0.148	0.26
**Trust**	4.12 (0.81)	4.24 (0.68)	3.20 (1.00)	<0.001	1.30	4.24 (0.58)	3.29 (1.04)	<0.001	1.41
**Subjective Norm**	4.17 (0.80)	4.30 (0.70)	3.18 (0.87)	<0.001	1.48	4.27 (0.67)	3.33 (0.91)	<0.001	1.32
**Benefits**	3.98 (0.73)	4.01 (0.60)	2.86 (0.78)	<0.001	1.73	4.12 (0.57)	3.32 (0.78)	<0.001	1.32
**Moral Norm**	3.79 (0.79)	3.99 (0.69)	2.79 (0.87)	<0.001	1.59	4.00 (0.68)	3.21 (0.94)	<0.001	1.09
**Concern**	3.08 (0.87)	3.19 (0.83)	3.96 (0.59)	<0.001	1.01	3.03 (0.77)	3.79 (0.67)	<0.001	1.01
**Necessity**	3.80 (0.96)	3.68 (1.00)	2.70 (0.73)	<0.001	1.06	3.83 (0.97)	3.31 (0.79)	0.002	0.55
**PHQ-2** **(depression)**	1.16 (1.38)	1.33 (1.45)	1.36 (1.54)	0.816	0.02	1.23 (1.38)	1.03 (1.52)	0.422	0.14
**GAD-2** **(anxiety)**	1.19 (1.41)	1.34 (1.38)	1.30 (1.49)	0.729	0.03	1.38 (1.35)	1.00 (0.97)	0.047	0.29

**Table 3:** Multivariable binary logistic regression analysis of vaccine hesitancy in unvaccinated participants (*n* = 503) and parental hesitancy in parents with children between 12 to 18 years old (*n* = 233).

**Variables**	**Unvaccinated (Vaccine Hesitancy)** ***n*** ** = 503**			**Parents with Children Aged** **12 to 18 Years (Parental Hesitancy)** ***n*** ** = 233**		
	**Odds** **Ratio**	**95% Confidence Interval**	***p*** **-Value**	**Odds** **Ratio**	**95% Confidence Interval**	***p*** **-Value**
**Gender**						
Male	0.650	0.318–1.328	0.237	7.610	1.523–38.031	0.013
Female	Ref. ^1^	–	–	Ref.	–	–
**Race**						
Chinese	Ref.	–	–	Ref.	–	–
Non–Chinese	0.531	0.138–2.048	0.358	2.105	0.304–14.563	0.451
**Age**						
17–30	2.335	0.789–6.911	0.125	0.057	0.000–19.759	0.337
31–40	Ref.	–	–	1.693	0.113–25.437	0.703
41–50	0.812	0.312–2.115	0.669	Ref.	–	–
51–60	1.811	0.548–5.988	0.330	0.198	0.033–1.190	0.077
61+	1.209	0.350–4.170	0.764	0.123	0.004–3.749	0.230
**Highest Education**						
Secondary Education or lower	1.159	0.400–3.363	0.785	0.219	0.027–1.788	0.156
Post-secondary education or higher	Ref.	–	–	Ref.	–	–
**Monthly Household Income**						
Less than $5000	1.301	0.601–2.815	0.504	3.688	0.438–31.028	0.230
$5,000–$12,999	Ref.	–	–	Ref.	–	–
More than $13,000	0.721	0.268–1.939	0.517	1.110	0.236–5.220	0.895
**Housing Type**						
1–3 room HDB	0.776	0.301–2.001	0.599	0.371	0.034–4.042	0.416
4–5 room HDB/Executive Apartment/DBSS/HUDC	Ref.	–	–	Ref.	–	–
Condominium/Landed Property	0.727	0.322–1.639	0.442	0.340	0.081–1.431	0.141
**Occupation**						
Employed	Ref.	–	–	Ref.	–	–
Schooling	0.247	0.053–1.141	0.073	28.648	0.099–8316.44	0.246
Self-employed	0.704	0.258–1.924	0.494	0.490	0.076–3.161	0.453
Not employed or schooling	2.121	0.773–5.822	0.144	3.268	0.533–20.051	0.201
**Daily Regular Contact**						
Less than 10 people	Ref.	–	–	Ref.	–	–
10–19 people	1.410	0.648–3.068	0.386	1.398	0.334–5.854	0.647
20–49 people	1.045	0.402–2.712	0.929	0.753	0.100–5.685	0.783
50 people or more	0.377	0.065–2.191	0.277	1.286	0.168–9.873	0.809
**Living with children aged** **0 to 12 years**						
No	Ref.	–	–	Ref.	–	–
Yes	1.457	0.595–3.567	0.410	0.325	0.076–1.395	0.130
**Living with youth aged** **12 to 18 years**						
No	Ref.	–	–	2.224	0.238–20.761	0.483
Yes	0.654	0.191–2.234	0.498	Ref.	–	–
**Living with people with poor health**						
No	Ref.	–	–	Ref.	–	–
Yes	0.305	0.117–0.798	0.015	1.089	0.213–5.561	0.918
**Living with people vulnerable to COVID-19**						
No	Ref.	–	–	Ref.	–	–
Yes	0.923	0.420–2.029	0.842	0.316	0.044–2.279	0.253
**Living with spouse**						
No	Ref.	–	–	0.851	0.117–6.158	0.873
Yes	0.765	0.323–1.815	0.544	Ref.	–	–
**Has a Chronic Condition**						
No	Ref.	–	–	Ref.	–	–
Yes	1.490	0.693–3.202	0.307	0.656	0.173–2.488	0.535
**GAD-2**	1.085	0.799–1.473	0.602	0.455	0.195–1.063	0.069
**PHQ-2**	0.978	0.718–1.331	0.886	0.828	0.449–1.526	0.545
**Perceived Risk of COVID-19**	1.622	0.922–2.853	0.094	0.241	0.074–0.785	0.018
**Trust**	1.004	0.592–1.703	0.987	0.350	0.091–1.347	0.127
**Subjective Norm**	0.374	0.237–0.592	<0.001	0.420	0.151–1.172	0.098
**Benefits**	0.249	0.109–0.569	0.001	0.147	0.025–0.875	0.035
**Moral Norm**	0.246	0.136–0.444	<0.001	0.641	0.181–2.268	0.490
**Concern**	1.429	0.757–2.697	0.271	6.309	1.800–22.113	0.004
**Necessity**	0.423	0.269–0.666	<0.001	4.317	1.461–12.758	0.008
**Vaccination Status**						
Vaccinated	–	–	–	Ref.	–	–
Unvaccinated/Have not completed full regimen	–	–	–	4.571	0.829–25.208	0.081

^1^ Reference group.

## Data Availability

The data presented in the study are available on request from the corresponding author.

## References

[1] Ministry of Health (MOH) Singapore Updates on Singapore’s COVID-19 Situation. https://www.moh.gov.sg/COVID–19.

[2] World Health Organisation (WHO) WHO Coronavirus (COVID-19) Dashboard. https://covid19.who.int/.

[3] Cihan P. (2021). Forecasting fully vaccinated people against COVID-19 and examining future vaccination rate for herd immunity in the US, Asia, Europe, Africa, South America, and the World. Appl. Soft Comput..

[4] Polack F.P., Thomas S.J., Kitchin N., Absalon J., Gurtman A., Lockhart S., Perez J.L., Marc G.P., Moreira E.D., Zerbini C. (2020). Safety and efficacy of the BNT162b2 mRNA COVID-19 vaccine. New Engl. J. Med..

[5] Voysey M., Clemens S.A.C., Madhi S.A., Weckx L.Y., Folegatti P.M., Aley P.K., Angus B., Baillie V.L., Barnabas S.L., Bhorat Q.E. (2021). Safety and efficacy of the ChAdOx1 nCoV-19 vaccine (AZD1222) against SARS-CoV-2: An interim analysis of four randomised controlled trials in Brazil, South Africa, and the UK. Lancet.

[6] Rodrigues C.M.C., Plotkin S.A. (2020). Impact of Vaccines; Health, Economic and Social Perspectives. Front. Microbiol..

[7] Hajj Hussein I., Chams N., Chams S., El Sayegh S., Badran R., Raad M., Gerges-Geagea A., Leone A., Jurjus A. (2015). Vaccines Through Centuries: Major Cornerstones of Global Health. Front. Public Health.

[8] Andre F.E., Booy R., Bock H.L., Clemens J., Datta S.K., John T.J., Lee B.W., Lolekha S., Peltola H., Ruff T.A. (2008). Vaccination greatly reduces disease, disability, death and inequity worldwide. Bull. World Health Organ..

[9] Ang H.M. NCID Nurse Becomes First Person in Singapore to Receive COVID-19 Vaccine. https://www.channelnewsasia.com/singapore/covid-19-first-vaccinations-ncid-healthcare-workers-pfizer-511886.

[10] Teo G. Singapore Starts Vaccinating Seniors against COVID-19 with Pilot Exercises in Tanjong Pagar, Ang Mo Kio. https://www.channelnewsasia.com/singapore/covid-19-vaccination-exercise-ang-mo-kio-tanjong-pagar-pm-lee-435576.

[11] Co C. COVID-19 Vaccination Now Open to Singapore Residents Aged 45 to 59. https://www.channelnewsasia.com/singapore/covid-19-vaccination-younger-age-groups-277536.

[12] Mahmud A.H. Singaporeans Aged 12 to 39 Can Register for COVID-19 Vaccination from Jun 11. https://www.channelnewsasia.com/singapore/covid-19-vaccination-age-12-to-39-recovered-single-dose-1846706.

[13] Ang H.M. COVID-19 Vaccinations for Singapore Students Aged 12 and above Begin. https://www.channelnewsasia.com/singapore/covid-19-vaccinations-singapore-students-pfizer-biontech-1828431.

[14] Abdullah A.Z. Pfizer–BioNTech COVID-19 Vaccine Approved by Singapore, First Shipment Expected by End–December. https://www.channelnewsasia.com/singapore/singapore-approves-pfizer-biontech-covid-19-vaccine-phase-3-478436.

[15] Lai L. S’pore Approves Moderna’s COVID-19 Vaccine; First Shipment to Arrive around March. https://www.straitstimes.com/singapore/health/spore-approves-modernas-covid-19-vaccine-first-shipment-to-arrive-around-march.

[16] Singapore M.O.H. FAQs-Safety and Efficacy of the COVID-19 Vaccine. https://www.moh.gov.sg/covid-19/vaccination/faqs---safety-and-efficacy-of-the-covid-19-vaccine.

[17] Tjendro J. Suspected Adverse Effects Reported in 0.12% of mRNA COVID-19 Vaccine Doses Administered: HSA. https://www.channelnewsasia.com/singapore/covid-19-vaccine-adverse-effects-pfizer-moderna–sinovac-2116006.

[18] MacDonald N.E., Eskola J., Liang X., Chaudhuri M., Dube E., Gellin B., Goldstein S., Larson H., Manzo M.L., Reingold A. (2015). Vaccine hesitancy: Definition, scope and determinants. Vaccine.

[19] Chirumbolo S. (2021). Vaccination hesitancy and the “myth” on mRNA-based vaccines in Italy in the COVID-19 era: Does urgency meet major safety criteria?. J. Med. Virol..

[20] Lazarus J.V., Ratzan S.C., Palayew A., Gostin L.O., Larson H.J., Rabin K., Kimball S., El-Mohandes A. (2021). A global survey of potential acceptance of a COVID-19 vaccine. Nat. Med..

[21] Gallè F., Sabella E.A., Roma P., De Giglio O., Caggiano G., Tafuri S., Da Molin G., Ferracuti S., Montagna M.T., Liguori G. (2021). Knowledge and Acceptance of COVID-19 Vaccination among Undergraduate Students from Central and Southern Italy. Vaccines.

[22] Nomura S., Eguchi A., Yoneoka D., Kawashima T., Tanoue Y., Murakami M., Sakamoto H., Maruyama-Sakurai K., Gilmour S., Shi S. (2021). Reasons for being unsure or unwilling regarding intention to take COVID-19 vaccine among Japanese people: A large cross–sectional national survey. Lancet Reg. Health-West. Pac..

[23] Schwarzinger M., Watson V., Arwidson P., Alla F., Luchini S. (2021). COVID-19 vaccine hesitancy in a representative working–age population in France: A survey experiment based on vaccine characteristics. Lancet Public Health.

[24] Paul E., Steptoe A., Fancourt D. (2021). Attitudes towards vaccines and intention to vaccinate against COVID-19: Implications for public health communications. Lancet Reg. Health-Eur..

[25] Khubchandani J., Sharma S., Price J.H., Wiblishauser M.J., Sharma M., Webb F.J. (2021). COVID-19 vaccination hesitancy in the United States: A rapid national assessment. J. Community Health.

[26] Stojanovic J., Boucher V.G., Gagne M., Gupta S., Joyal-Desmarais K., Paduano S., Aburub A.S., Sheinfeld Gorin S.N., Kassianos A.P., Ribeiro P.A.B. (2021). Global trends and correlates of COVID-19 vaccination hesitancy: Findings from the icare study. Vaccines.

[27] Soares P., Rocha J.V., Moniz M., Gama A., Laires P.A., Pedro A.R., Dias S., Leite A., Nunes C. (2021). Factors associated with COVID-19 vaccine hesitancy. Vaccines.

[28] Wang J., Jing R., Lai X., Zhang H., Lyu Y., Knoll M.D., Fang H. (2020). Acceptance of COVID-19 vaccination during the COVID-19 pandemic in China. Vaccines.

[29] Machida M., Nakamura I., Kojima T., Saito R., Nakaya T., Hanibuchi T., Takamiya T., Odagiri Y., Fukushima N., Kikuchi H. (2021). Acceptance of a COVID-19 vaccine in Japan during the COVID-19 pandemic. Vaccines.

[30] Lin C., Tu P., Beitsch L.M. (2021). Confidence and receptivity for COVID-19 vaccines: A rapid systematic review. Vaccines.

[31] Harapan H., Wagner A.L., Yufika A., Winardi W., Anwar S., Gan A.K., Setiawan A.M., Rajamoorthy Y., Sofyan H., Mudatsir M. (2020). Acceptance of a COVID-19 Vaccine in Southeast Asia: A Cross-Sectional Study in Indonesia. Front. Public Health.

[32] Bell S., Clarke R., Mounier–Jack S., Walker J.L., Paterson P. (2020). Parents’ and guardians’ views on the acceptability of a future COVID-19 vaccine: A multi–methods study in England. Vaccine.

[33] Troiano G., Nardi A. (2021). Vaccine hesitancy in the era of COVID-19. Public Health.

[34] Dror A.A., Eisenbach N., Taiber S., Morozov N.G., Mizrachi M., Zigron A., Srouji S., Sela E. (2020). Vaccine hesitancy: The next challenge in the fight against COVID-19. Eur. J. Epidemiol..

[35] Wong M.C.S., Wong E.L.Y., Huang J., Cheung A.W.L., Law K., Chong M.K.C., Ng R.W.Y., Lai C.K.C., Boon S.S., Lau J.T.F. (2021). Acceptance of the COVID-19 vaccine based on the health belief model: A population–based survey in Hong Kong. Vaccine.

[36] Chen H., Li X., Gao J., Liu X., Mao Y., Wang R., Zheng P., Xiao Q., Jia Y., Fu H. (2021). Health belief model perspective on the control of COVID-19 vaccine hesitancy and the promotion of vaccination in China: Web–based cross–sectional study. J. Med. Internet Res..

[37] Al–Metwali B.Z., Al–Jumaili A.A., Al–Alag Z.A., Sorofman B. (2021). Exploring the acceptance of COVID-19 vaccine among healthcare workers and general population using health belief model. J. Eval. Clin. Pract..

[38] Mercadante A.R., Law A.V. (2021). Will they, or Won’t they? Examining patients’ vaccine intention for flu and COVID-19 using the Health Belief Model. Res. Soc. Adm. Pharm..

[39] Lin Y., Hu Z., Zhao Q., Alias H., Danaee M., Wong L.P. (2020). Understanding COVID-19 vaccine demand and hesitancy: A nationwide online survey in China. PLoS Negl. Trop. Dis..

[40] Wong L.P., Alias H., Wong P.F., Lee H.Y., AbuBakar S. (2020). The use of the health belief model to assess predictors of intent to receive the COVID-19 vaccine and willingness to pay. Hum. Vaccines Immunother..

[41] Prickett K.C., Habibi H., Carr P.A. (2021). COVID-19 Vaccine Hesitancy and Acceptance in a Cohort of Diverse New Zealanders. Lancet Reg. Health–West. Pac..

[42] Sowa P., Kiszkiel Ł., Laskowski P.P., Alimowski M., Szczerbiński Ł., Paniczko M., Moniuszko-Malinowska A., Kamiński K. (2021). COVID-19 vaccine hesitancy in Poland—multifactorial impact trajectories. Vaccines.

[43] Lim V.W., Lim R.L., Tan Y.R., Soh A.S.E., Tan M.X., Othman N.B., Dickens S.B., Thein T.L., Lwin M.O., Ong R.T.H. (2021). Government trust, perceptions of COVID-19 and behaviour change: Cohort surveys, singapore. Bull. World Health Organ..

[44] World Health Organisation (WHO) Report of the SAGE Working Group on Vaccine Hesitancy. https://www.who.int/immunization/sage/meetings/2014/october/1_Report_WORKING_GROUP_vaccine_hesitancy_final.pdf.

[45] Painter J.E., Borba C.P.C., Hynes M., Mays D., Glanz K. (2008). The use of theory in health behavior research from 2000 to 2005: A systematic review. Ann. Behav. Med..

[46] Davis R., Campbell R., Hildon Z., Hobbs L., Michie S. (2015). Theories of behaviour and behaviour change across the social and behavioural sciences: A scoping review. Health Psychol. Rev..

[47] Glanz K., Bishop D.B. (2010). The Role of Behavioral Science Theory in Development and Implementation of Public Health Interventions. Annu. Rev. Public Health.

[48] Goldman R.D., Yan T.D., Seiler M., Parra Cotanda C., Brown J.C., Klein E.J., Hoeffe J., Gelernter R., Hall J.E., Davis A.L. (2020). Caregiver willingness to vaccinate their children against COVID-19: Cross sectional survey. Vaccine.

[49] Alfieri N.L., Kusma J.D., Heard–Garris N., Davis M.M., Golbeck E., Barrera L., Macy M.L. (2021). Parental COVID-19 vaccine hesitancy for children: Vulnerability in an urban hotspot. BMC Public Health.

[50] Ministry of Health (MOH) Singapore Updates on Local Situation and Heightened Alert to Minimise Transmission (14 May 2021). https://www.moh.gov.sg/news-highlights/details/updates-on-local-situation-and-heightened-alert-to-minimise-transmission-14May.

[51] Ministry of Health (MOH) Singapore COVID-19 Situation Report. https://covidsitrep.moh.gov.sg/.

[52] Ministry of Health (MOH) Singapore Update on Local COVID-19 Situation (31 July). https://www.moh.gov.sg/news–highlights/details/update-on-local-covid-19-situation-(31-july).

[53] National Population and Talent Division (NPTD) Singapore Population Trends-Overview. https://www.population.gov.sg/our-population/population-trends/overview.

[54] Singstats (2021). Population Trends, 2021.

[55] Kroenke K., Spitzer R.L., Janet B.W.W. (2003). The Patient Health Questionnaire-2: Validity of a Two-Item Depression Screener. Med. Care.

[56] Arroll B., Goodyear-Smith F., Crengle S., Gunn J., Kerse N., Fishman T., Falloon K., Hatcher S. (2010). Validation of PHQ-2 and PHQ-9 to Screen for Major Depression in the Primary Care Population. Ann. Fam. Med..

[57] Plummer F., Manea L., Trepel D., McMillan D. (2016). Screening for anxiety disorders with the GAD-7 and GAD-2: A systematic review and diagnostic metaanalysis. Gen. Hosp. Psychiatry.

[58] Spitzer R.L., Kroenke K., Williams J.B.W., Löwe B. (2006). A Brief Measure for Assessing Generalized Anxiety Disorder: The GAD-7. Arch. Intern. Med..

[59] Becker M.H. (1974). The health belief model and personal health behavior. Health Educ. Monogr..

[60] Jones C.L., Jensen J.D., Scherr C.L., Brown N.R., Christy K., Weaver J. (2015). The Health Belief Model as an Explanatory Framework in Communication Research: Exploring Parallel, Serial, and Moderated Mediation. Health Commun..

[61] Ajzen I. (1991). The theory of planned behavior. Organ. Behav. Hum. Decis. Process..

[62] Ursachi G., Horodnic I.A., Zait A. (2015). How Reliable are Measurement Scales? External Factors with Indirect Influence on Reliability Estimators. Procedia Econ. Financ..

[63] Choi S.H., Jo Y.H., Jo K.J., Park S.E. (2021). Pediatric and Parents’ Attitudes Towards COVID-19 Vaccines and Intention to Vaccinate for Children. J. Korean Med. Sci..

[64] Rhodes A., Hoq M., Measey M.A., Danchin M. (2021). Intention to vaccinate against COVID-19 in Australia. Lancet Infect. Dis..

[65] Yan Z., Yang M., Lai C.L. (2021). COVID-19 vaccinations: A comprehensive review of their safety and efficacy in special populations. Vaccines.

[66] Kwok K.O., Li K.-K., Wei W.I., Tang A., Wong S.Y.S., Lee S.S. (2021). Influenza vaccine uptake, COVID-19 vaccination intention and vaccine hesitancy among nurses: A survey. Int. J. Nurs. Stud..

[67] Fernandes N., Costa D., Costa D., Keating J., Arantes J. (2021). Predicting COVID-19 Vaccination Intention: The Determinants of Vaccine Hesitancy. Vaccines.

[68] Bavel J.J.V., Baicker K., Boggio P.S., Capraro V., Cichocka A., Cikara M., Crockett M.J., Crum A.J., Douglas K.M., Druckman J.N. (2020). Using social and behavioural science to support COVID-19 pandemic response. Nat. Hum. Behav..

[69] Graupensperger S., Abdallah D.A., Lee C.M. (2021). Social norms and vaccine uptake: College students’ COVID vaccination intentions, attitudes, and estimated peer norms and comparisons with influenza vaccine. Vaccine.

[70] Ruggiero K.M., Wong J., Sweeney C.F., Avola A., Auger A., Macaluso M., Reidy P. (2021). Parents’ Intentions to Vaccinate Their Children Against COVID-19. J. Pediatric Health Care.

[71] Wang Q., Xiu S., Zhao S., Wang J., Han Y., Dong S., Huang J., Cui T., Yang L., Shi N. (2021). Vaccine hesitancy: COVID-19 and influenza vaccine willingness among parents in wuxi, China—A cross-sectional study. Vaccines.

[72] Napolitano F., D’Alessandro A., Angelillo I.F. (2018). Investigating Italian parents’ vaccine hesitancy: A cross–sectional survey. Hum. Vaccines Immunother..

[73] Wang Y., Hunt K., Nazareth I., Freemantle N., Petersen I. (2013). Do men consult less than women? An analysis of routinely collected UK general practice data. BMJ Open.

[74] Scaife B., Gill P.S., Heywood P.L., Neal R.D. (2000). Socio-economic characteristics of adult frequent attenders in general practice: Secondary analysis of data. Fam. Pract..

[75] Pomara C., Sessa F., Ciaccio M., Dieli F., Esposito M., Giammanco G.M., Garozzo S.F., Giarratano A., Prati D., Rappa F. (2021). COVID-19 Vaccine and Death: Causality Algorithm According to the WHO Eligibility Diagnosis. Diagnostics.

[76] Rodeghiero F., Balduini C.L. (2021). A new enemy is emerging in the fight against the SARS-CoV-2 pandemic. Haematologica.

[77] Schneider J., Sottmann L., Greinacher A., Hagen M., Kasper H.U., Kuhnen C., Schlepper S., Schmidt S., Schulz R., Thiele T. (2021). Postmortem investigation of fatalities following vaccination with COVID-19 vaccines. Int. J. Leg. Med..

